# Deoxycholic Acid and Coronary Artery Calcification in the Chronic Renal Insufficiency Cohort

**DOI:** 10.1161/JAHA.121.022891

**Published:** 2022-03-24

**Authors:** Anna Jovanovich, Xuan Cai, Rebecca Frazier, Josh D. Bundy, Jiang He, Panduranga Rao, Claudia Lora, Mirela Dobre, Alan Go, Tariq Shafi, Harold I. Feldman, Eugene P. Rhee, Makoto Miyazaki, Tamara Isakova, Michel Chonchol, the CRIC Study Investigators, Lawrence J. Appel, Jing Chen, James P. Lash, Robert G. Nelson, Mahboob Rahman, Panduranga S Rao, Vallabh O. Shah, Raymond R. Townsend, Mark L. Unruh

**Affiliations:** ^1^ Renal Section VA Eastern Colorado Healthcare System Aurora CO; ^2^ Division of Renal Diseases and Hypertension University of Colorado Anschutz Medical Campus Aurora CO; ^3^ Division of Nephrology/Hypertension Northwestern University Chicago IL; ^4^ Nephrology and Hypertension Tulane University New Orleans LA; ^5^ Division of Nephrology University of Michigan Ann Arbor MI; ^6^ Division of Nephrology University of Illinois at Chicago Chicago IL; ^7^ Division of Nephrology Case Western Reserve University Cleveland OH; ^8^ Division of Research Kaiser Permanente Northern California Oakland CA; ^9^ Division of Nephrology University of Mississippi Jackson MI; ^10^ Division of Renal Electrolyte and Hypertension University of Pennsylvania Philadelphia PA; ^11^ Nephrology Division Massachusetts General Hospital Harvard Medical School Boston MA

**Keywords:** chronic kidney disease, coronary artery calcification, deoxycholic acid, microbiome, secondary bile acid, Vascular Disease

## Abstract

**Background:**

Deoxycholic acid (DCA) is a secondary bile acid that may promote vascular calcification in experimental settings. Higher DCA levels were associated with prevalent coronary artery calcification (CAC) in a small group of individuals with advanced chronic kidney disease. Whether DCA levels are associated with CAC prevalence, incidence, and progression in a large and diverse population of individuals with chronic kidney disease stages 2 to 4 is unknown.

**Methods and Results:**

In the CRIC (Chronic Renal Insufficiency Cohort) study, we evaluated cross‐sectional (n=1057) and longitudinal (n=672) associations between fasting serum DCA levels and computed tomographic CAC using multivariable‐adjusted regression models. The mean age was 57±12 years, 47% were women, and 41% were Black. At baseline, 64% had CAC (CAC score >0 Agatston units). In cross‐sectional analyses, models adjusted for demographics and clinical factors showed no association between DCA levels and CAC >0 compared with no CAC (prevalence ratio per 1‐SD higher log DCA, 1.08 [95% CI, 0.91–1.26). DCA was not associated with incident CAC (incidence per 1‐SD greater log DCA, 1.08 [95% CI, 0.85–1.39]) or CAC progression (risk for increase in ≥100 and ≥200 Agatston units per year per 1‐SD greater log DCA, 1.05 [95% CI, 0.84–1.31] and 1.26 [95% CI, 0.77–2.06], respectively).

**Conclusions:**

Among CRIC study participants, DCA was not associated with prevalent, incident, or progression of CAC.

**Nonstandard Abbreviations and Acronyms**
CRIC: Chronic Renal Insufficiency Cohort
CYP7A1: cholesterol 7‐alpha‐hydroxylase
DCA: deoxycholic acid

**Clinical Perspective**

**What Is New?**

- This is the first analysis of a large, diverse cohort of individuals with chronic kidney disease that aimed to determine whether circulating levels of the bile acid, deoxycholic acid, were associated with vascular calcification.
- Despite preclinical data suggesting that deoxycholic acid is directly toxic to vascular smooth muscle cells causing vascular calcification, and a small cross‐sectional observational study demonstrating a significant association between higher circulating deoxycholic acid levels and vascular calcification, we found no association between higher deoxycholic acid levels and prevalence, incidence, and progression of coronary artery calcification among participants in the CRIC (Chronic Renal Insufficiency Cohort) study.

**What Are the Clinical Implications?**

- The bile acid, deoxycholic acid, should not be used as a predictor of vascular calcification among individuals with chronic kidney disease stages 2 to 4.
- Further preclinical and clinical research is required to more precisely determine the role deoxycholic acid and other bile acids play in vascular calcification and adverse outcomes in chronic kidney disease.

Vascular calcification is common in chronic kidney disease (CKD).^1^, ^2^, ^3^ Sixty‐six percent of CRIC (Chronic Renal Insufficiency Cohort) study participants demonstrate moderate to severe coronary artery calcification (CAC).^3^, ^4^ Vascular calcification is associated with arterial stiffness, which contributes to left ventricular hypertrophy and CKD progression,^5^, ^6^ and it is also independently associated with cardiovascular disease events and mortality in CKD.^7^ It is imperative to better understand the pathophysiology of vascular calcification to identify biomarkers that predict presence and severity and determine treatment targets to prevent and slow its progression.

Bile acids are produced in hepatocytes by CYP7A1 (cholesterol 7‐alpha‐hydroxylase) and conjugated with either taurine or glycine. Conjugated bile acids are then secreted into the bile canaliculi and stored in the gallbladder. From the gallbladder, bile acids are excreted into the intestinal lumen where their main function is to emulsify dietary lipids. Once in the intestinal lumen, primary bile acids undergo bacteria‐induced transformation to secondary bile acids.^8^ More than 95% of bile acids are excreted in stool, and the remainder is reabsorbed into the circulation by the ileum.^9^ In addition to their role in lipid digestion, bile acids are involved in lipid and glucose metabolism via interaction with the nuclear FXR (farnesoid X receptor),^8^, ^10^ which is found in the liver, kidneys, intestines, macrophages, and vasculature, including vascular endothelial cells^11^ and vascular smooth muscle cells.^12^

Deoxycholic acid (DCA) is a secondary bile acid derived from gut bacterial transformation of the primary bile acid, cholic acid. Among individuals with CKD, DCA levels are higher compared with those with normal kidney function.^13^, ^14^ Although some preclinical work demonstrates DCA is directly toxic to vascular smooth muscle cells and induces vascular calcification through endoplasmic reticulum stress,^14^ other work finds DCA does not cause vascular smooth muscle cell calcification.^15^ Our group previously demonstrated elevated serum DCA levels are independently associated with greater CAC scores in a small cross‐sectional analysis of individuals with moderate to severe CKD (mean estimated glomerular filtration rate [eGFR], 32±9 mL/min per 1.73 m^2^).^16^ Because DCA production is dependent on the composition of gut bacteria, CKD‐associated gut microbiome dysbiosis^17^ may be responsible for increased DCA levels observed in CKD. Diet and medications can change the composition of the microbiome, suggesting that DCA levels may be modifiable and a potential treatment target to reduce vascular calcification, cardiovascular disease, and premature mortality in CKD.

The CRIC study is a longitudinal observational study and includes a large, diverse sample of participants with CKD stages 2 to 4. We tested the hypothesis that higher circulating DCA levels would be associated with greater CAC prevalence, incidence, and progression.

## Methods

Anonymized data and materials have been made publicly available at the CRIC National Institute of Diabetes and Digestive and Kidney Diseases biorepository and can be accessed at https://repository.niddk.nih.gov/studies/cric/.

### Study Design and Participants

The CRIC study is a prospective cohort study of a racially and ethnically diverse group of men and women aged 21 to 74 years with mild to moderate CKD (eGFR entry criteria, 20–70 mL/min per 1.73 m^2^). A total of 3939 participants were enrolled from 7 centers in the United States between May 2003 and August 2008.^18^ Individuals who were unable to consent, institutionalized, enrolled in other studies, pregnant, had New York Heart Association class III to IV heart failure, human immunodeficiency virus infection, cirrhosis, myeloma, polycystic kidney disease, renal cancer, recent chemotherapy or immunosuppressive therapy, those receiving maintenance dialysis, or an organ transplant were excluded. The study was approved by the institutional review boards from each clinical center, and all participants provided written informed consent.

### Computed Tomographic Measurements

**Figure 1:**
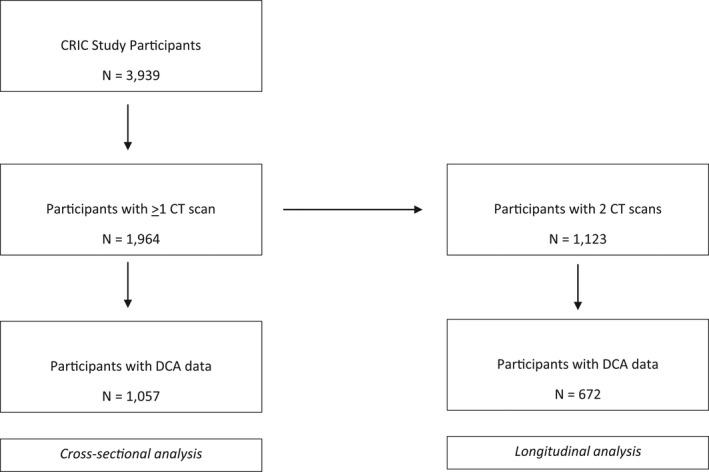
Selection of participants for final analytic cohort. CRIC indicates Chronic Renal Insufficiency Cohort; CT, computed tomography; and DCA, deoxycholic acid.

Participants with a history of coronary artery revascularization did not undergo computed tomography (CT) measurements. Of those with no history of coronary artery revascularization, 1142 participants were randomly selected and stratified by age, sex, race and ethnicity, diabetes status, and eGFR for electron‐beam or multidetector CT. In addition, all eligible participants from 3 centers were scanned as part of an ancillary study, yielding 1964 total participants scanned within the first 3 years of the original baseline examination. Of these participants, 1057 had fasting DCA levels measured at the same study visit as their first CT scan (ie, baseline for the present study) as part of an ancillary study. Repeat CT was performed among 1123 participants an average±SD of 3.2±0.6 years later, 672 of whom had DCA data (Figure 1).

Trained and certified technologists scanned participants twice using phantoms of known physical calcium concentrations. One cardiologist read all scans at a central reading center (Los Angeles Biomedical Research Institute at Harbor‐University of California, Los Angeles Medical Center) to quantify calcification according to the Agatston score.^19^ Total CAC score was calculated as the sum of scores from the left main, left anterior descending, left circumflex, and right coronary arteries. Final scores are the mean of 2 scans.^20^

### Exposure Assessment

Fasting serum samples stored at −80 °C were shipped with sufficient dry ice to the University of Colorado for measurement of DCA using liquid chromatography–tandem mass spectrometry, as previously described.^21^ In brief, human serum (100 µL) was diluted in 300 µL of cold acetonitrile containing 30 ng of D6‐DCA (Cambridge Isotope Laboratory) as internal standard. The mixture was passed through a Phree phospholipid removal plate (Phenomenex). The eluate was evaporated with nitrogen gas stream, then redissolved in 100 µL of 10 mmol/L ammonium acetate buffer (pH 8.0)/methanol (1:1, v/v). A 10‐µL aliquot of each sample solution was then analyzed with Applied Biosystems 3200 qTRAP DC/MS/MS (SCIEX). The intra‐assay coefficient of variation for the DCA measurements was 4.3%. Fifteen percent of the DCA values were undetectable, defined by the laboratory as <4 ng/mL. These undetectable results were replaced with the value 2 ng/mL, half of the lower limit of detection.^22^, ^23^

### Covariate Assessment

We obtained covariate data from the same study visit as the first CT scan or the most recent previous annual visit if missing. Self‐reported sociodemographic characteristics, medical history, and current medications were obtained using a questionnaire. Body weight, height, and blood pressure were measured using standard protocols.^18^ Diabetes was defined as fasting glucose level ≥126 mg/dL, nonfasting glucose level ≥200 mg/dL, and/or use of antidiabetic medications. History of cardiovascular disease was defined as self‐reported prior coronary artery disease, heart failure, stroke, or peripheral vascular disease.

Glucose, cholesterol, phosphate, calcium, magnesium, serum albumin, and total parathyroid hormone were measured using standard laboratory methods. The 24‐hour urinary protein was measured using the turbidometric method with benzethonium chloride. FGF23 (fibroblast growth factor 23) was measured using a second‐generation carboxy‐terminal assay (Immutopics). hs‐CRP (high‐sensitivity C‐reactive protein) and IL‐6 (interleukin‐6) were measured at the original baseline examination using the particle‐enhanced immunonephelometry method. We calculated eGFR using the Chronic Kidney Disease Epidemiology Collaboration equation.^24^

### Statistical Analysis

We summarized baseline participant characteristics as mean±SD or median with interquartile range for continuous variables and percentages for categorical variables by DCA tertile. Statistical differences between tertiles were tested using ANOVA for continuous variables with normal distributions, Wilcoxon‐Mann‐Whitney test for continuous variables with skewed distributions, and χ^2^ tests for categorical variables. We evaluated the cross‐sectional association of circulating DCA level with CAC score using a 2‐part model.^20^ First, we modeled the prevalence of CAC score >0 among all participants using logistic regression. Second, among those with CAC score >0, we modeled the severity of CAC (ie, ≥100 and ≥400 units) using logistic regression. Regression coefficients were expressed as CAC prevalence ratios per 1‐SD higher log‐transformed DCA or between tertiles of DCA compared with the lowest tertile (reference).

We evaluated the longitudinal association of DCA with CAC stratifying by the presence of baseline CAC.^25^ Among those with no baseline CAC (CAC=0 Agatston units), we defined the incidence as CAC score >0 at follow‐up. Among those with baseline CAC (CAC >0 Agatston units), we assessed progression defined as an annual increase in CAC score ≥100 units, which is significantly associated with higher risk for coronary heart disease.^26^ Additionally, we assessed progression defined as an annual increase in CAC score ≥200 units. We evaluated the association between DCA and CAC incidence and progression using Poisson regression with robust variance estimation, using an offset to account for the time between CT scans. To determine whether DCA was associated with any CAC progression (as opposed to a cut point of CAC score ≥100 units or ≥200 units), we used linear regression analysis and CAC as a continuous variable defined as mean annualized change in CAC.

We included covariates in sequential regression models based on prior clinical knowledge. In addition to unadjusted analyses, 4 multivariable‐adjusted models were used: (1) adjusted for age, sex, race, ethnicity, and clinical site; (2) adjusted for variables in model 1 plus eGFR, 24‐hour urinary protein, diabetes, systolic blood pressure, number of antihypertensive medications, current smoking, history of cardiovascular disease, total cholesterol level, and use of statin medications; (3) adjusted for variables in model 2 plus hs‐CRP and IL‐6; and (4) adjusted for variables in model 3 plus albumin, calcium, phosphate, magnesium, parathyroid hormone, and FGF23. Because the onset of end‐stage kidney disease may increase the risk for calcification, we conducted sensitivity analyses excluding those with end‐stage kidney disease at baseline (ie, at the time of the scan, cross‐sectional analyses) and during follow‐up (longitudinal analyses).

We tested for effect modification by including DCA‐by‐subgroup interaction terms (defined by age, sex, race and ethnicity, diabetes, total cholesterol, eGFR, and statin use) in logistic regression and Poisson regression models. All analyses were conducted using SAS version 9.4 (SAS Institute). All tests were 2‐sided, and statistical significance was defined as *P*<0.05.

## Results

Among 1057 CRIC participants with data for DCA and CAC obtained by CT, the mean age was 57±12 years, 47% were women, 45% had diabetes, 28% had self‐reported history of cardiovascular disease, and mean eGFR was 43±17 mL/min per 1.73 m^2^. Median (interquartile range) DCA was 65 ng/mL (23–137 ng/mL). As shown in Table 1, individuals with higher circulating DCA levels were older (*P*=0.001), had higher eGFR (*P*=0.007) and lower urinary protein excretion (*P*=0.03), and lower circulating FGF23 levels (*P*=0.008) compared with individuals with lower DCA levels.

**Table 1:** Baseline Characteristics of 1057 Chronic Renal Insufficiency Cohort Participants by Tertiles of DCA

	Tertile 1 (DCA ≤33 ng/mL), n=349	Tertile 2 (DCA 34–106 ng/mL), n=349	Tertile 3 (DCA >106 ng/mL), n=359	*P* value
Age, y	56±12	56±12	59±11	0.001
Women, %	48	45	47	0.71
Black, %	43	41	39	0.51
Hispanic, %	5	5	5	0.94
BMI, kg/m^2^	31±7	30±7	32±7	0.13
Smoking, %	13	11	9	0.20
CVD, %	29	26	29	0.55
Diabetes, %	48	41	45	0.14
Antihypertensive, n	2.4±1.3	2.4±1.3	2.4±1.3	0.61
SBP, mm Hg	125±22	126±21	126±20	0.89
Cholesterol, mg/dL	184±44	183±41	183±42	0.96
Statin use, %	59.3	53.0	55.2	0.23
eGFR, mL/min per 1.73 m^2^	41±17	45±17	44±16	0.007
Urinary protein, g/24 h	0.23 (0.08–1.08)	0.15 (0.07–0.82)	0.14 (0.06–0.89)	0.03
Serum albumin, g/dL	4.0±0.5	4.1±0.4	4.1±0.4	0.02
IL‐6, pg/mL	1.70 (1.07–2.57)	1.58 (0.95–2.51)	1.76 (1.11–2.87)	0.69
CRP, mg/L	2.41 (0.96–5.46)	1.83 (0.76–4.57)	2.15 (0.89–5.55)	0.28
Magnesium, mg/dL	1.93±0.30	1.94±0.28	1.92±0.26	0.58
Calcium, mg/dL	9.3±0.5	9.3±0.5	9.3±0.6	0.16
Phosphate, mg/dL	3.9±1.2	3.8±1.0	3.8±0.7	0.40
FGF23, RU/mL	157 (100–314)	132 (93–276)	129 (86–231)	0.008
PTH, pg/mL	63 (41–105)	62 (40–97)	61 (42–93)	0.78

Results are reported as proportions, mean±standard deviation, or median (interquartile range). Covariate data are from visit 5. If covariate data were missing at visit 5, they were obtained from visit 3. BMI indicates body mass index; CRP, C‐reactive protein; CVD, cardiovascular disease; DCA, deoxycholic acid; eGFR, estimated glomerular filtration rate; FGF23, fibroblast growth factor 23; IL‐6, interleukin 6; PTH, parathyroid hormone; RU, reference units; and SBP, systolic blood pressure.

**Figure 2:**
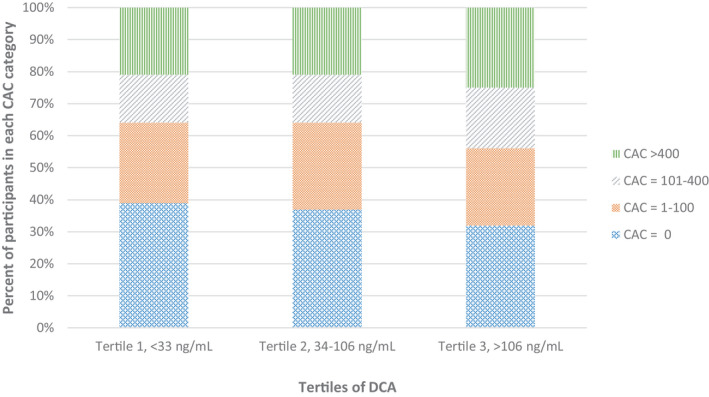
Distribution of the percentage of participants in each baseline CAC category by tertile of DCA. DCA is presented as nanograms per milliliter and CAC as Agatston units. CAC indicates coronary artery calcification; and DCA, deoxycholic acid.

Figure 2 shows the distribution of baseline CAC by DCA tertiles: ≤33 ng/mL, 34 to 106 ng/mL, and >106 ng/mL. Compared with participants with DCA levels in the lowest tertile, participants with DCA levels in the highest tertile had the lowest prevalence of CAC=0 and had slightly greater CAC prevalence at 101 to 400 Agatston units and at >400 Agatston units (*P*=0.30).

Table 2 shows cross‐sectional associations of DCA with baseline CAC prevalence and severity. Of 1057 participants, 676 (64%) had baseline CAC (CAC >0 Agatston units). In unadjusted analyses, compared with DCA level in the lowest tertile, DCA levels in the highest tertile were associated with greater CAC prevalence (prevalence ratio, 1.37 [95% CI, 1.01–1.87]). However, after adjustment for demographics, clinical characteristics, including markers of kidney disease and cardiovascular disease, measures of inflammation, and mineral metabolites, the association between DCA and prevalent CAC was no longer statistically significant (adjusted prevalence ratio, 1.30 [95% CI, 0.87–1.94]).

**Table 2:** Results of Logistic Regression Analysis Showing the Cross‐Sectional Association of Baseline DCA With Prevalence and Severity of Baseline CAC

Prevalence of CAC (any Agatston score >0), prevalence ratio (95% CI), all participants, n=1057				
No. of events/No. of participants	Per 1‐SD increase log DCA* 676/1057	Tertile 1 DCA ≤33 212/349	Tertile 2 DCA 34–106 220/349	Tertile 3 DCA >106 244/359
Unadjusted	1.09 (0.96–1.23)	Reference	1.10 (0.81–1.50)	1.37 (1.01–1.87)
Model 1	0.98 (0.85–1.14)	Reference	1.01 (0.71–1.43)	1.03 (0.72–1.48)
Model 2	1.08 (0.92–1.26)	Reference	1.21 (0.82–1.77)	1.27 (0.86–1.87)
Model 3	1.09 (0.93–1.28)	Reference	1.24 (0.84–1.83)	1.33 (0.90–1.98)
Model 4	1.08 (0.91–1.26)	Reference	1.18 (0.80–1.75)	1.30 (0.87–1.94)

CAC severity (any Agatston score ≥100 units or ≥400 units) among participants with prevalent baseline CAC (any Agatston score >0), prevalence ratio (95% CI), total n=676				
Prevalence of CAC ≥100 Agatston units, prevalence ratio (95% CI)				
Events/total	Per 1‐SD increase log DCA* 405/676	Tertile 1 DCA ≤36 124/212	Tertile 2 DCA 37–112 125/220	Tertile 3 DCA >112 156/244
Unadjusted	1.09 (0.93–1.27)	Reference	0.93 (0.64–1.37)	1.26 (0.86–1.84)
Model 1	1.06 (0.90–1.25)	Reference	0.96 (0.64–1.45)	1.13 (0.75–1.70)
Model 2	1.15 (0.96–1.37)	Reference	1.22 (0.79–1.89)	1.40 (0.91–2.16)
Model 3	1.16 (0.97–1.39)	Reference	1.20 (0.77–1.88)	1.42 (0.92–2.21)
Model 4	1.14 (0.95–1.37)	Reference	1.21 (0.76–1.91)	1.34 (0.85–2.11)

Prevalence of CAC ≥400 Agatston units, prevalence ratio (95% CI)				
Events/total	Per 1‐SD increase log DCA* 236/676	Tertile 1 DCA ≤36 74/212	Tertile 2 DCA 37–112 73/220	Tertile 3 DCA >112 89/244
Unadjusted	0.98 (0.83–1.15)	Reference	0.93 (0.62–1.38)	1.07 (0.73–1.57)
Model 1	0.93 (0.79–1.11)	Reference	0.99 (0.65–1.51)	0.95 (0.63–1.43)
Model 2	1.01 (0.84–1.21)	Reference	1.22 (0.77–1.93)	1.14 (0.73–1.76)
Model 3	1.01 (0.84–1.21)	Reference	1.20 (0.76–1.91)	1.13 (0.73–1.76)
Model 4	0.998 (0.83–1.21)	Reference	1.20 (0.74–1.94)	1.11 (0.70–1.76)

Model 1: adjusted for age, sex, race, ethnicity, and clinical site. Model 2: model 1 plus eGFR, 24‐hour urinary protein, diabetes, SBP, number of antihypertensive medications, current smoking, history of CVD, total cholesterol, and statin use. Model 3: model 2 plus IL‐6 and CRP. Model 4: model 3 plus PTH, FGF23, phosphate, calcium, albumin, and magnesium. Covariate data are from visit 5. If covariate data were missing at visit 5, they were obtained from visit 3. CAC indicates coronary artery calcification; CRP, C‐reactive protein; CVD, cardiovascular disease; DCA, deoxycholic acid; eGFR, estimated glomerular filtration rate; FGF23, fibroblast growth factor 23; IL‐6, interleukin 6; PTH, parathyroid hormone; and SBP, systolic blood pressure.

* For the continuous analysis, the prevalence ratio 1.09 (95% CI, 0.96–1.23) is interpreted as a 9% (95% CI, −0.04% to 23%) increase in CAC prevalence for every 1‐SD increase in log DCA level.

Of 676 participants with prevalent CAC (CAC >0 Agatston units), 405 (60%) had CAC ≥100 Agatston units and 236 (35%) had severe CAC defined as CAC ≥400 Agatston units. In unadjusted analyses, there was no association between DCA in the highest tertile compared with the lowest tertile and CAC ≥100 Agatston units (prevalence ratio, 1.26 [95% CI, 0.86–1.84]), nor was there any association in the multivariable‐adjusted model (adjusted prevalence ratio, 1.34 [95% CI, 0.85–2.11]) (Table 2). Similarly, there was no association between DCA in the highest tertile compared with the lowest tertile and severe CAC (≥400 Agatston units) in either the unadjusted (prevalence ratio, 1.07 [95% CI, 0.73–1.57]) or the adjusted (adjusted prevalence ratio, 1.11 [95% CI, 0.70–1.76]) models (Table 2). Likewise, when DCA was treated as a continuous variable, there was no association with prevalence or severity of CAC per 1‐SD greater log DCA (Table 2).

Table 3 shows longitudinal associations of DCA with CAC incidence and progression among 672 participants who had a repeat CT scan a mean of 3.2±0.6 years after the baseline CT scan. Among 277 participants with no baseline CAC (CAC <0 Agatston units), 60 (22%) developed incident CAC during follow‐up. In unadjusted analyses, there was no association between baseline DCA in the highest tertile compared with the lowest tertile and incident CAC (relative risk, 1.28 [95% CI, 0.76–2.15]). The lack of association remained after multivariable adjustment (relative risk, 1.22 [95% CI, 0.71–2.08]). Among 395 participants with baseline CAC (CAC >0 Agatston units), 78 (20%) had an annual increase of ≥100 Agatston units and 29 (7%) had an annual increase of ≥200 Agatston units. In unadjusted analyses, there was no association between baseline DCA in the highest tertile compared with the lowest tertile and progression of CAC with an annual increase of ≥100 Agatston units (relative risk, 1.36 [95% CI, 0.84–2.20]). The lack of association remained after multivariable adjustment (relative risk, 1.26 [95% CI, 0.76–2.11]). Similarly, there was no association between baseline DCA in the highest tertile compared with the lowest tertile and progression of CAC with an annual increase of ≥200 Agatston units in the unadjusted analyses (relative risk, 1.95 [95% CI, 0.81–4.68]). After multivariable adjustment, baseline DCA in the highest tertile compared with the lowest tertile was associated with >2‐fold higher risk of CAC progression ≥200 Agatston units per year (relative risk, 2.64 [95% CI, 1.14–6.15]). When baseline DCA was treated as a continuous variable, there was no association with CAC incidence or progression per 1‐SD greater in log DCA in either the unadjusted or fully adjusted models. Finally, there was no association between baseline CAC and mean annualized change in CAC (Table S1).

**Table 3:** Results of Poisson Regression Analysis to Determine the Longitudinal Association of Baseline DCA With CAC Incidence and Results of Linear Regression Analysis to Determine Progression of CAC Among Chronic Renal Insufficiency Cohort Study Participants With Baseline and Follow‐Up Computed Tomography Scan

Incident CAC progression among participants with no baseline CAC (Agatston score=0), n=277				
No. of events/No. of participants	Per 1‐SD increase log DCA* 60/277	Tertile 1 DCA ≤29 19/92	Tertile 2 DCA 30–94 16/90	Tertile 3 DCA >94 25/95
Unadjusted	1.09 (0.85–1.39)	Reference	0.83 (0.46–1.50)	1.28 (0.76–2.15)
Model 1	1.03 (0.80–1.33)	Reference	0.85 (0.47–1.54)	1.14 (0.69–1.87)
Model 2	1.06 (0.83–1.34)	Reference	1.00 (0.57–1.75)	1.16 (0.70–1.93)
Model 3	1.05 (0.82–1.34)	Reference	1.03 (0.59–1.79)	1.16 (0.70–1.94)
Model 4	1.08 (0.85–1.39)	Reference	1.09 (0.62–1.90)	1.22 (0.71–2.08)

CAC progression among participants with baseline CAC (Agatston score >0), n=395				
CAC progression of ≥100 Agatston units/year, relative risk (95% CI)				
Events/total	Per 1‐SD increase log DCA^†^ 78/395	Tertile 1 DCA ≤34 23/130	Tertile 2 DCA 35–102 23/131	Tertile 3 DCA >102 32/134
Unadjusted	1.14 (0.91–1.44)	Reference	0.97 (0.57–1.65 )	1.36 (0.84–2.20)
Model 1	1.06 (0.86–1.30)	Reference	1.11 (0.66–1.88)	1.18 (0.72–1.93)
Model 2	1.09 (0.88–1.35)	Reference	1.31 (0.78–2.21)	1.35 (0.82–2.23)
Model 3	1.07 (0.86–1.33)	Reference	1.15 (0.68–1.96)	1.31 (0.80–2.16)
Model 4	1.05 (0.84–1.31)	Reference	1.06 (0.62–1.83)	1.26 (0.76–2.11)

CAC progression of ≥200 Agatston units/year, relative risk (95% CI)				
Events/total	Per 1‐SD increase log DCA^†^ 29/395	Tertile 1 DCA ≤34 7/130	Tertile 2 DCA 35–102 8/131	Tertile 3 DCA >102 14/134
Unadjusted	1.31 (0.83–2.08)	Reference	1.11 (0.41–2.98)	1.95 (0.81–4.68)
Model 1	1.18 (0.75–1.85)	Reference	1.25 (0.45–3.51)	1.60 (0.61–4.17)
Model 2	1.18 (0.77–1.81)	Reference	1.32 (0.50–3.48)	1.91 (0.86–4.22)
Model 3	1.17 (0.75–1.82)	Reference	1.21 (0.44–3.32)	1.93 (0.87–4.30)
Model 4	1.26 (0.77–2.06)	Reference	1.75 (0.54–5.67)	2.64 (1.14–6.15)

Model 1: adjusted for age, sex, race, ethnicity, clinical site, baseline CAC (among those with CAC >0 only). Model 2: model 1 plus eGFR, 24‐hour urinary protein, diabetes, SBP, number of antihypertensive medications, current smoking, history of CVD, total cholesterol, and statin use. Model 3: model 2 plus IL‐6 and CRP. Model 4: model 3 plus PTH, FGF23, phosphate, calcium, albumin, and magnesium. Covariate data are from visit 5. If covariate data were missing at visit 5, they were obtained from visit 3. CAC indicates coronary artery calcification; CRP, C‐reactive protein; CVD, cardiovascular disease; DCA, deoxycholic acid; eGFR, estimated glomerular filtration rate; FGF23, fibroblast growth factor 23; IL‐6, interleukin 6; PTH, parathyroid hormone; and SBP, systolic blood pressure.

* For the continuous analysis, the relative risk of incident CAC 1.09 (95% CI, 0.85–1.39) is interpreted as a 9% (95% CI, −0.15% to 39%) increase in CAC incidence for every 1‐SD increase in log DCA level among Chronic Renal Insufficiency Cohort study participants without CAC at baseline.

^†^ For the continuous analysis, the relative risk of progressive CAC, 1.14 (95% CI, 0.91–1.44) is interpreted as a 14% (95% CI, −0.09% to 44%) increase in CAC progression of ≥100 Agatston units/year for every 1‐SD increase in log DCA level among Chronic Renal Insufficiency Cohort study participants with CAC at baseline.

We detected a borderline significant interaction between age and DCA among those with prevalent CAC ≥400 Agatston units (*P*=0.045) and a statistically significant interaction between diabetes and DCA among participants (n=29) with an increase in CAC of ≥200 Agatston units per year (*P*=0.004). We did not explore these interactions further because of the borderline significant *P* value and small number of participants, which limited power. Furthermore, we did not detect any statistically significant interaction between DCA and age, sex, race and ethnicity, diabetes, statin use, eGFR, or cholesterol level in the other analyses (*P*>0.05 for all). Sensitivity analyses excluding participants with end‐stage kidney disease did not affect results (Table S2).

## Discussion

DCA is a secondary bile acid derived from the primary bile acid, cholic acid, via intestinal bacteria transformation. Among individuals with CKD, DCA levels are higher compared with those with normal kidney function.^13^, ^14^ In preclinical experiments, DCA, but not other bile acids, induced vascular smooth muscle calcification through endoplasmic reticulum stress,^14^ and DCA was independently associated with CAC among a group of individuals with moderate to severe CKD.^16^ Despite these compelling preclinical and observational data supporting a role for excess circulating DCA in the pathophysiology of vascular calcification in CKD, in this analysis of 1057 CRIC participants with mean eGFR 43±17 mL/min per 1.73 m^2^, circulating DCA levels were not associated with prevalent CAC in cross‐sectional analyses nor with incident CAC or CAC progression in longitudinal analyses.

Bile acid handling in CKD may be dysregulated. Various small observational studies suggest circulating bile acid levels are elevated in CKD.^13^, ^14^, ^27^, ^28^ A small observational study of 61 patients with CKD (mean±SD creatinine, 2.96±0.77 mg/dL), found higher circulating bile acid levels significantly correlated with lower eGFR, leading the investigators to conclude that reduced urinary excretion of bile acids led to elevated blood levels in CKD.^28^ However, evidence from animal models suggests that bile acid transport^29^ or production^14^ may be altered in CKD. Additionally, the proportion of DCA compared with its precursor, cholic acid, is elevated in CKD.^13^ Because intestinal bacteria are responsible for the biotransformation of cholic acid to DCA, it is also plausible that CKD‐associated alterations in the gut microbiome may influence DCA and other bile acid levels. Based on these observations, we expected DCA levels to be higher among individuals with lower GFR. However, in this analysis we found the opposite; CRIC participants with higher DCA levels in the second and third tertiles had better kidney function, eGFR of 45±17 and 44±16 mL/min per 1.73 m^2^, respectively, compared with participants with lower DCA levels in the first tertile (eGFR, 41±17 mL/min per 1.73 m^2^). Mechanisms underlying possible bile acid metabolism dysregulation in kidney disease require further investigation.

Bile acid metabolism is implicated in vascular disease, including vascular calcification.^11^, ^12^, ^21^, ^30^, ^31^, ^32^, ^33^ Activation of FXR inhibits vascular calcification in an animal model of CKD^15^ and reduces atherosclerotic plaque formation in other non‐CKD animal models.^21^, ^33^ Activation of FXR also reduces levels of circulating DCA.^21^ Taken together, these observations suggest FXR activation and lower circulating DCA levels may prevent or attenuate vascular calcification. DCA, but not other bile acids, induced calcification in cultured bovine vascular smooth muscle cells, and these cells overexpressed the ATF‐4 (activation transcription factor 4) and CHOP (C/EBP homologous protein) pathways, indicating endoplasmic reticulum stress as the mechanism for calcification.^14^ Oxidative stress is another factor implicated in vascular calcification.^34^ Excess bile acids, including DCA, induce cytotoxicity through oxidative stress.^35^ Endothelial cells incubated in DCA demonstrated increased reactive oxygen species, which induced cellular pathways leading to monocyte adhesion, an early sign of vascular dysfunction and atherosclerotic lesions.^36^ However, not all published experimental evidence demonstrates DCA, and other bile acids cause vascular calcification. Human vascular smooth muscle cells did not calcify when incubated in either DCA or lithocholic acid.^15^ Likewise, vascular calcification was not more severe in an animal model of progressive CKD fed a DCA‐supplemented diet compared with animals with progressive CKD fed a control diet.^15^

Our finding that DCA is not independently associated with CAC prevalence, incidence, or progression in a large group of CRIC participants is contrary to a prior report,^16^ which evaluated the cross‐sectional relationship between DCA and presence of CAC among 112 individuals with moderate to advanced CKD (mean eGFR, 31.5±8.7 mL/min per 1.73 m^2^) and found an independent association between higher log‐transformed DCA and greater CAC volume score.^16^ A potential explanation for these conflicting findings is different population characteristics. Compared with the prior report,^16^ CRIC participants were younger by ≈10 years, had less severe kidney disease (mean eGFR, 41±17 mL/min per 1.73 m^2^ in CRIC versus mean eGFR 31.5±8.7 mL/min per 1.73 m^2^ in the prior report), and a lower burden of vascular calcification (in the CRIC study, >50% had CAC volume scores ≤100 Agatston units and 36% had no CAC at baseline [Figure 2], whereas in the prior report, the median [interquartile range] CAC volume score was 246 [43–743] Agatston units). However, age, kidney function, and calcification burden do not seem to modify the relationship between DCA and CAC scores. We did not find any interaction between age and DCA nor eGFR and DCA on CAC scores, and there was no association between DCA and CAC severity in the CRIC study. In the prior report,^16^ only 112 individuals were available for the cross‐sectional analysis, and although the confidence intervals were narrow, the small sample size may not have provided enough power to reject the null hypothesis. The CRIC study includes data from a racially and geographically diverse group of individuals with a broad range of kidney function (CKD stages 2–4). We analyzed over 1000 participants for the cross‐sectional analysis, and 672 participants for the longitudinal analyses. This large and diverse sample size engenders confidence in the conclusion that there is no relationship between DCA and CAC in CKD.

Our study has several strengths. First, it is the first to evaluate DCA as a predictor of CAC prevalence, incidence, and progression in a large, diverse cohort of mild to moderate CKD. Second, the CRIC study uses standardized methods and measurements of CAC and other variables across clinical sites, which minimizes bias. Third, we were able to adjust for numerous covariates including markers of mineral metabolism and inflammation, which are known contributors to vascular calcification in CKD. Notwithstanding these strengths, there are also limitations. First, we were not able to measure DCA levels over time; thus, we do not know how circulating levels vary over time among this large group of individuals with CKD. Second, we do not have measures of other bile acids so cannot draw conclusions about the total bile acid pool or bile acid ratios. Third, CRIC did not collect stool samples, and thus we were unable to measure DCA in the stool. Among individuals without CKD, stool bile acids are a more validated marker of atherosclerosis than circulating bile acids.^37^, ^38^ Fourth, it is plausible there is a relationship between DCA and CAC progression when CAC progression is defined by an annual increase <100 Agatston units. We defined CAC progression as an annual increase in CAC ≥100 Agatston units based on previous data,^26^ because an annual increase in CAC ≥100 Agatston units is associated with clinical outcomes (coronary heart disease events) in CKD.^25^ Nonetheless, there was no significant association between baseline DCA and mean annualized change in CAC. Finally, the event numbers in the progression analysis were fairly small; nonetheless, this is still the largest and most comprehensive study evaluating the association of circulating DCA and CAC.

In conclusion, DCA was not associated with CAC prevalence, incidence, or progression among a large, diverse cohort of individuals with CKD stages 2 to 4. Further experimental and clinical research is required to more precisely determine the role DCA and other bile acids play in vascular calcification and adverse outcomes in CKD.

## Appendix

### CRIC Study Investigators

Lawrence J. Appel, MD, MPH; Jing Chen, MD, MMSc, MSc; James P. Lash, MD; Robert G. Nelson, MD, PhD, MS; Mahboob Rahman, MD; Panduranga S Rao, MD; Vallabh O. Shah, PhD, MS; Raymond R. Townsend, MD; Mark L. Unruh, MD, MS.

## Sources of Funding

This work was supported by the George M. O’Brien Kidney Research Center at Northwestern University (NU‐GoKIDNEY; P30DK114857) from the National Institutes of Health (NIH)/National Institute of Diabetes and Digestive and Kidney Diseases (NIDDK). A.J. is supported b**y** Veterans Affairs Merit Award I01CX001985. R.F. is supported by grant T32DK108738 from the NIDDK. T.I. is supported by grants R01DK102438, R01DK110087, and U01DK099930 from the NIDDK and K24HL150235 from the National Heart, Lung, and Blood Institute. Funding for the CRIC study was obtained under a cooperative agreement from the National Institute of Diabetes and Digestive and Kidney Diseases (U01DK060990, U01DK060984, U01DK061022, U01DK061021, U01DK061028, U01DK060980, U01DK060963, U01DK060902 and U24DK060990). In addition, this work was supported in part by the Perelman School of Medicine at the University of Pennsylvania Clinical and Translational Science Award NIH/NCATS UL1TR000003, Johns Hopkins University UL1 TR‐000424, University of Maryland GCRC M01 RR‐16500, Clinical and Translational Science Collaborative of Cleveland, UL1TR000439 from the National Center for Advancing Translational Sciences (NCATS) component of the NIH and NIH roadmap for Medical Research, Michigan Institute for Clinical and Health Research (MICHR) UL1TR000433, University of Illinois at Chicago CTSA UL1RR029879, Tulane COBRE for Clinical and Translational Research in Cardiometabolic Diseases P20 GM109036, Kaiser Permanente NIH/NCRR UCSF‐CTSI UL1 RR‐024131, Department of Internal Medicine, University of New Mexico School of Medicine Albuquerque, NM R01DK119199.

## Disclosures

Dr Dobre reports receiving consultancy fees from Tricia and Relypsa. Dr Shafi reports receiving an honorarium from Siemens for a continuing medical education lecture. Dr Feldman reports consulting fees from Kyowa Hakko Kirini Co. Ltd, is the Editor‐in‐Chief of the *American Journal of Kidney Disease*, coaches for InMed Physicians, and works for DLA Piper LLP for Essure Litigation. Dr Isakova reports receiving consulting fees from Akebia Therapeutics. Dr Chonchol reports grants from Otsuka, Sanofi, and Kadmon. The remaining authors have no disclosures to report.

## Supporting information

Tables S1–S2
