# Supplementary material for: Deoxycholic Acid and Coronary Artery Calcification in the Chronic Renal Insufficiency Cohort
Source: J Am Heart Assoc. 2022 Mar 24;11(7):e022891. doi: 10.1161/JAHA.121.022891 (PMC9075491; doi:10.1161/JAHA.121.022891)
Supplement: Supplementary file 1 — Tables S1–S2 [file JAH3-11-e022891-s001.pdf]

SUPPLEMENTAL MATERIAL

Table S1. Longitudinal association of baseline DCA with mean annualized change in CAC.

Participants with baseline and follow-up CAC scores, N = 672				
Mean annualized change in CAC (95% CI)				
	Per 1-SD increase log DCA	Tertile 1 DCA ≤29	Tertile 2 DCA 30 – 94	Tertile 3 DCA >94
Unadjusted	34.5 (5.7 – 63.3)	33.2 (21.0 – 45.3)	36.8 (0.5 – 73.2)	45.2 (8.9 – 81.4)
Model 1	34.4 (1.6 – 67.2)	33.1 (21.0 – 45.2)	36.6 (0.3 – 73.0)	45.1 (8.9 – 81.3)
Model 2	34.4 (1.5 – 67.3)	33.1 (20.9 – 45.2)	36.8 (-3.3 – 76.8)	45.7 (5.8 – 85.7)
Model 3	34.3 (-29.3 – 97.9)	33.1 (20.8 – 45.5)	36.6 (-3.9 – 77.1)	46.4 (5.9 – 86.8)
Model 4	35.0 (0.4 – 69.7)	34.2 (21.4 – 47.0)	36.1 (-16.2 – 88.4)	47.5 (-5.0 – 100.1)

Model 1: adjusted for age, sex, race, ethnicity, clinical site, baseline CAC (among those with CAC >0 only).

Model 2: model 1 plus eGFR, 24-hour urinary protein, diabetes, SBP, number of antihypertensive medications, current smoking, history of CVD, total cholesterol, and statin use.

Model 3: model 2 plus IL-6 and CRP.

Model 4: model 3 plus PTH, FGF23, phosphate, calcium, albumin, and magnesium.

DCA, deoxycholic acid; SBP, systolic blood pressure; eGFR, estimated glomerular filtration rate; PTH, parathyroid hormone; FGF23, fibroblast growth factor 23; RU, reference units; CRP, c-reactive protein; IL-6, Interleukin 6.

Table S2. Impact of excluding participants with end-stage renal disease on associations of DCA with prevalence and severity of CAC at baseline, and incidence and progression of CAC.

Per 1-SD increase log DCA	All Participants			Participants without ESRD		
	Sample Size	Estimate (95% CI)	*p* -Value	Sample Size	Estimate (95% CI)	*p* -Value
**Cross-sectional**						
All Participants						
CAC >0, Prevalence Ratio	1057	1.08 (0.91 – 1.26)	0.38	1045	1.07 (0.91 – 1.26)	0.42
Baseline CAC>0						
CAC ≥100, Prevalence Ratio	676	1.14 (0.95 – 1.37)	0.16	669	1.14 (0.95 – 1.37)	0.16
CAC ≥400, Prevalence Ratio	676	0.998 (0.83 – 1.21)	0.98	669	0.995 (0.82 – 1.20)	0.96
**Longitudinal**						
Baseline CAC=0						
Incident CAC, Relative Risk	277	1.08 (0.85 – 1.39)	0.52	244	1.15 (0.84 – 1.57)	0.37
Baseline CAC>0						
Increase ≥100 Agatston units/year, Relative Risk	395	1.05 (0.84 – 1.31)	0.66	359	1.02 (0.81 – 1.29)	0.86

Increase ≥200 Agatston units/year, Relative Risk	395	1.26 (0.77 – 2.06)	0.36	359	1.39 (0.82 – 2.35)	0.22

Adjusted for age, sex, race, ethnicity, clinical site, baseline CAC (among those with CAC >0 only), eGFR, 24-hour urinary protein, diabetes, SBP, number of antihypertensive medications, current smoking, history of CVD, total cholesterol, statin use, IL-6, CRP, PTH, FGF23, phosphate, calcium, albumin, and magnesium.

DCA, deoxycholic acid; SBP, systolic blood pressure; eGFR, estimated glomerular filtration rate; PTH, parathyroid hormone; FGF23, fibroblast growth factor 23; RU, reference units; CRP, c-reactive protein; IL-6, Interleukin 6.

Covariate data are from visit 5. If covariate data were missing at visit 5, they were obtained from visit 3.
